# Microglia deficiency accelerates prion disease but does not enhance prion accumulation in the brain

**DOI:** 10.1002/glia.24244

**Published:** 2022-07-19

**Authors:** Barry M. Bradford, Lynne I. McGuire, David A. Hume, Clare Pridans, Neil A. Mabbott

**Affiliations:** ^1^ The Roslin Institute and R(D)SVS University of Edinburgh, Easter Bush Campus Midlothian UK; ^2^ Mater Research Institute‐University of Queensland, Translational Research Institute Woolloongabba Queensland Australia; ^3^ Simons Initiative for the Developing Brain, Centre for Discovery Brain Sciences University of Edinburgh, Hugh Robson Building Edinburgh UK; ^4^ Centre for Inflammation Research The Queen's Medical Research Institute, Edinburgh BioQuarter Edinburgh UK

**Keywords:** central nervous system, microglia, neurodegeneration, prion disease, reactive astrocyte

## Abstract

Prion diseases are transmissible, neurodegenerative disorders associated with misfolding of the prion protein. Previous studies show that reduction of microglia accelerates central nervous system (CNS) prion disease and increases the accumulation of prions in the brain, suggesting that microglia provide neuroprotection by phagocytosing and destroying prions. In *Csf1r* ^ΔFIRE^ mice, the deletion of an enhancer within *Csf1r* specifically blocks microglia development, however, their brains develop normally and show none of the deficits reported in other microglia‐deficient models. *Csf1r* ^ΔFIRE^ mice were used as a refined model in which to study the impact of microglia‐deficiency on CNS prion disease. Although *Csf1r* ^ΔFIRE^ mice succumbed to CNS prion disease much earlier than wild‐type mice, the accumulation of prions in their brains was reduced. Instead, astrocytes displayed earlier, non‐polarized reactive activation with enhanced phagocytosis of neuronal contents and unfolded protein responses. Our data suggest that rather than simply phagocytosing and destroying prions, the microglia instead provide host‐protection during CNS prion disease and restrict the harmful activities of reactive astrocytes.

## Main Points

- CNS prion disease is accelerated in mice completely lacking microglia.
- The rate of prion accumulation in the brain was unaltered in absence of microglia.
- Microglia provide host‐protection during CNS prion disease independent of prion clearance.

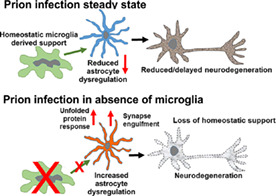

## 1 INTRODUCTION

The parenchymal macrophages of the central nervous system (CNS) are known as microglia (Hortega, 1919) and their proliferation and survival is dependent upon signaling via the colony stimulating factor 1 receptor (CSF1R) (Hume et al., 2020). Microglia have been attributed essential functions in the development and homeostasis of the CNS including synaptogenesis, neurogenesis, and maturation of neuronal circuits (Prinz et al., 2019). However, mice with a *Csf1r* hypomorphic mutation (*Csf1r* ^ΔFIRE^) (Rojo et al., 2019), with conditional *Csf1r* deletion (using Iba1‐cre) (Nakayama et al., 2018) and rats with a *Csf1r* null mutation (Pridans et al., 2018) each lack microglia entirely but have normal CNS development. These findings indicate that developmental roles of microglia are redundant as studies reveal their functions can be carried out by other cells when microglia are absent (Damisah et al., 2020; Guo et al., 2019; Patkar et al., 2021). There is much greater evidence that microglia contribute to neuropathology (Prinz et al., 2019). Neurodegenerative diseases associated with mutations in microglia‐expressed genes such as *CSF1R* in humans have been referred to as microgliopathies (Hume et al., 2020).

Prion diseases, or transmissible spongiform encephalopathies, are fatal progressive neurodegenerative diseases to which there are no cures. Infectious prions are considered to result from the misfolding of the host's cellular prion protein (PrP^C^) into an abnormal disease‐associated isoform (PrP^Sc^) (Prusiner, 1982). The accumulation of PrP^Sc^ within the brain is accompanied by the impairment of neuronal dendritic spines and synapse structures, glial cell activation, vacuolar (spongiform) degeneration and ultimately neurodegeneration. Inhibiting the proliferation and pro‐inflammatory responses of microglia via CSF1R inhibition decelerated CNS prion disease (Gómez‐Nicola et al., 2013). Conversely, the partial depletion or deficiency in microglia was reported to enhance the accumulation of prions in the brain and accelerate the onset of clinical disease (Carroll et al., 2018; Zhu et al., 2016). However, none of these studies resulted in 100% microglial ablation nor addressed the potential confounding effects of ablative cell death or bystander effects, such as impact upon other non‐microglial CSF1R‐sensitive mononuclear phagocyte populations. For example although the CSF1R‐targeting kinase inhibitor PLX5622 has been widely used to ablate the microglia in the brain, such kinase inhibitors also impact peripheral CSF1R‐dependent macrophages (Hume & Macdonald, 2012). Since the ablation of peripheral macrophages enhances prion accumulation in the secondary lymphoid tissues (Beringue et al., 2000; Maignien et al., 2005), effects on peripheral macrophage populations in the above studies also cannot be excluded.

To address the above concerns we investigated CNS prion disease in *Csf1r* ^ΔFIRE^ mice which have a complete and specific lack of microglia in the brain but retain brain‐associated macrophages (Rojo et al., 2019). We show that microglial‐deficiency in *Csf1r* ^ΔFIRE^ mice was associated with accelerated prion disease in the absence of increased PrP^Sc^ accumulation or prion‐seeding activity. Instead, earlier astrocyte activation was associated with increased engulfment of neuronal contents and unfolded protein responses without induction of genes associated with neurotoxic (A1) or neuroprotective (A2) reactive astrocyte polarization (Liddelow et al., 2017). These data indicate that microglia provide neuroprotection during CNS prion disease independently of PrP^Sc^ clearance, and restrict the harmful effects of reactive astrocyte activation. Identification of the mechanisms by which the microglia provide neuroprotection during CNS prion disease may reveal novel targets for therapeutic intervention in these and other neurodegenerative disorders.

## 2 MATERIALS AND METHODS

### 2.1 Ethics statement

Ethical approvals for the in vivo mouse experiments were obtained from The Roslin Institute's and University of Edinburgh's ethics committees. These experiments were also performed under the authority of a UK Home Office Project License and in accordance with the guidelines and regulations of the UK Home Office “Animals (scientific procedures) Act 1986.” Appropriate care was provided to minimize harm and suffering, and anesthesia was administered where necessary. Mice were humanely culled at the end of the experiments by cervical dislocation.

### 2.2 Mice

*Csf1r* ^ΔFIRE/WT^ mice produced in‐house (Rojo et al., 2019) were crossed to produce homozygous *Csf1r* ^ΔFIRE^ (*Csf1r* ^ΔFIRE/ΔFIRE^) or *Csf1r* ^WT^ (*Csf1r* ^WT/WT^) littermates. Offspring were genotyped as described (Rojo et al., 2019). Pups were weaned and co‐housed under specific pathogen‐free conditions. Food and water were provided ad libitum.

### 2.3 Prion infection

Mice were infected at 10 weeks old via intracerebral injection with 20 μl of a 1.0% (wt/vol) brain homogenate prepared from mice terminally infected with ME7 scrapie prions. Mice were culled at the intervals indicated after exposure, or observed for signs of clinical prion disease as described elsewhere (Brown & Mabbott, 2014) and culled at a standard clinical end‐point. Survival times were calculated as the interval between injection and positive clinical assessment of terminal prion disease. Groups of age‐matched *Csf1r* ^ΔFIRE^ mice and *Csf1r* ^WT^ mice were used throughout the study.

### 2.4 Gait analysis

Gait analysis was performed weekly using the CatWalkXT (Noldus) from 8 weeks of age until positive clinical assessment of prion disease. Uninfected mice of both genotype were monitored weekly from 8 to 30 weeks of age as controls.

### 2.5 Neuropathological analysis

Clinical prion disease diagnosis was confirmed by histopathological assessment of vacuolation (spongiform pathology) in the brain. Coronal sections of paraffin‐embedded brain tissue were cut at 6 μm thickness, de‐paraffinized, and stained with hematoxylin & eosin and scored for spongiform vacuolar degeneration as described previously (Fraser & Dickinson, 1967). For the construction of lesion profiles, sections were scored for the presence and severity (scale 0–5) of prion‐disease‐specific vacuolation in nine gray matter and three white matter areas: G1, dorsal medulla; G2, cerebellar cortex; G3, superior colliculus; G4, hypothalamus; G5, thalamus; G6, hippocampus; G7, septum; G8, retrosplenial and adjacent motor cortex; G9, cingulate and adjacent motor cortex; W1, inferior and middle cerebellar peduncles; W2, decussation of superior cerebellar peduncles; and W3, cerebellar peduncles.

### 2.6 Immunohistochemistry

Paraffin‐embedded sections (thickness 6 μm) were deparaffinized, pre‐treated by autoclaving in distilled water at 121°C for 15 min, and for PrP‐immunostaining immersed in 98% formic acid for 10 min, endogenous peroxidases were quenched by immersion in 4% H_2_O_2_ in methanol for 5 min. Sections were incubated overnight with primary antibodies (see Table 1). Primary antibody binding was detected using biotinylated goat anti‐species specific antibodies (Jackson Immunoresearch, West Grove, PA) where necessary and visualized using the Elite ABC/HRP kit (Vector Laboratories, Peterborough, UK) and diaminobenzidine (DAB) between stringent washing steps. Sections were lightly counterstained with hematoxylin and imaged on a Nikon Ni.1 Brightfield Compound upright microscope, 4×/10×/20×/air lenses, Zeiss 105c color camera & Zen 2 software for image capture. For fluorescence immunohistochemistry primary antibodies were detected with species‐specific Alexa‐Fluor 488 or 594 conjugated secondary antibodies. Phosphorylated PERK (PERK‐P) staining was detected using biotinylated goat anti‐rabbit specific antibodies (Jackson Immunoresearch, West Grove, PA) and visualized using the Elite ABC/HRP kit (Vector Laboratories, Peterborough, UK) and Tyramide Alexa‐Fluor488 (Biotium) and imaged on a Zeiss LSM 710 Confocal Microscope with 6 Laser Lines (405/458/488/514/543/633 nm)/2 PMT's + 32 channel Quasar detector. 10×/20×/40 × 1.3 na oil/60 × 1.4na oil lenses using Zen Software.

**TABLE 1:** Primary antibodies

Target	Antibody	Supplier/reference
β‐Actin	Mouse monoclonal C4	Santa Cruz Biotechnology
C3	Rat monoclonal RMC11H9	Connex
CD44	Biotinylated rat anti‐mouse/human monoclonal IM7	Biolegend
eIF2a	Mouse monoclonal L57A5	Cell Signaling Technology
Gephryin	Mouse monoclonal 45/Gephryin	BD Biosciences
GFAP	Rabbit anti bovine polyclonal	Dako
Iba1	Rabbit polyclonal	Wako
Lcn2	Goat polyclonal	R&D Systems
NeuN	Mouse monoclonal A60	Millipore
PERK	Rabbit monoclonal C33E10	Cell Signaling Technology
Phopsho‐Eif2a (Ser51)	Rabbit monoclonal 119A11	Cell Signaling Technology
Phospho‐PERK (Thr980)	Rabbit monoclonal 16F8	Cell Signaling Technology
PrP	Mouse monoclonal BH1	McCutcheon et al. (2014)
PrP	Mouse monoclonal 6H4	Prionics
PSD95	Goat polyclonal	Abcam

### 2.7 Western blot analysis

Brain homogenates (10% wt/vol) were prepared in NP40 lysis buffer (1% NP40, 0.5% sodium deoxycholate, 150 mM NaCl, 50 mM TrisHCl [pH 7.5]). For the detection of PrP^Sc^ a sample of homogenate was incubated at 37°C for 1 h with 20 μg/ml proteinase K (PK) and digestion halted by addition of 1 mM phenylmethylsulfonyl fluoride. Samples were denatured at 98°C for 15 min in 1× SDS sample buffer (Life Technologies) and separated via electrophoresis through 12% Tris‐glycine polyacrylamide gels (Nupage, Life Technologies) and transferred to polyvinylidene difluoride PVDF membranes by semi‐dry electroblotting. Primary antibodies (Table 1) were detected by horseradish peroxidase‐conjugated goat anti‐species specific antibody (Jackson Immunoresearch) and visualized via chemiluminescence (BM Chemiluminescent substrate kit, Roche, Burgess Hill, UK) as described previously (Bradford et al., 2017).

### 2.8 Image analyses

Image analysis was performed using ImageJ software (http://imagej/nih.gov/ij) (Schneider et al., 2012). The magnitude of PrP^d^, GFAP, and CD44 immunostaining on DAB stained sections was compared as previously described (Bradford et al., 2019). Briefly, the optical density (OD) values for immunostaining were calculated using ImageJ software following H‐DAB deconvolution. Mean gray OD values were measured from DAB grayscale images (scaled 0–255) and expressed as a % relative intensity by dividing by the maximum value (255). Immunofluorescent images were analyzed using ImageJ as previously described (McCulloch et al., 2011). Briefly intensity thresholds were applied and then the number of pixels of each color counted and presented as a proportion of the total pixel area under analysis (% area coverage). The preferential co‐localization of fluorochromes was determined as previously described (McCulloch et al., 2011) by comparing the observed distribution of colors with those predicted by the null hypothesis that each element of positive staining was randomly and independently distributed. Values significantly greater (*P* < .05) than the null hypothesis confirm significant co‐localization of fluorochromes. The assessment of relative synaptic protein phagocytosis was calculated as the % of PSD95 or gephyrin staining co‐localized with GFAP relative to total of each synpaptic protein. Western blot images were subjected to densitometric analyzed by ImageJ. Briefly lanes and bands were identified, threshold levels set and area under the curve measurements taken (pixels). For PrP^C^ and PrP^Sc^ relative expression levels were calculated as a percentage relative to a control normal brain PrP^C^ measurement.

### 2.9 Real‐time quaking induced conversion (RT‐QuiC)

Brain homogenates were diluted at 10^–3^ vol/vol in PBS. RT‐QuIC reaction mix prepared as follows: 10 mM phosphate buffer (pH 7.4), 170 mM NaCl (total 300 mM including phosphate buffer), 0.1 mg/ml recombinant PrPc (Bank Vole 23‐230, [Orrú et al., 2015] construct kindly provided by Byron Caughey, Rockey Mountain Laboratories, Montana, USA), 10 μM Thioflavin‐T (ThT), and 10 μM ethylenediaminetetraacetic acid tetrasodium salt (EDTA). Reactions were performed in quadruplicate. Aliquots of the reaction mix (98 μl) were loaded into each well of a black 96‐well plate with a clear bottom (Thermo Scientific) and seeded with 2 μl of diluted brain homogenate. Samples were incubated in a FLUOstar® OMEGA plate reader (BMG LABTECH Ltd.) at 42°C for 80 h with intermittent shaking cycles: 1 min shake (double orbital, 700 rpm), 1 min rest. Fluorescence measurements (450 nm excitation and 480 nm emission; bottom read), referred to as relative fluorescent units (rfu) were taken every 15 min. A baseline rfu of ~38,000 for unseeded and initial BH seeded reactions were recorded, with saturation occurring at 260,000 rfu. All 4 quadruplicates of the 8 test samples, displayed a significant rise in rfu over time; a sample was considered “positive for PrP seeding” if replicates crossed a threshold of fluorescence set at 50,000 rfu based on the mean ± 10 *SD* (36,941 ± 8348) of the unseeded negative control samples analyzed. The mean time for each quadruplicate reading to reach the 50,000 rfu threshold was calculated and plotted.

### 2.10 Gene expression analysis via RT‐qPCR

Total RNA was isolated from brain using RNABee (AMSBio, Abingdon, UK) and RNeasy Mini kit (Qiagen). RNA was Dnase treated (Promega) to remove genomic DNA. Reverse transcription of polyA mRNA from 5 μg total DNA‐free RNA was performed using Superscript First Strand Synthesis (Invitrogen) with Oligo‐dT primers. Quantitative PCR (qPCR) were performed using SYBR master mix (Rox) (Roche) on an MX3005pro (Stratagene) using the primer sequences detailed (Table 2). Gene expression relative to naïve *Csf1r* ^WT^ mice was calculated using the ΔΔCT method (Livak & Schmittgen, 2001) using *Rpl19* as a reference gene.

**TABLE 2:** Oligonucleotide primers

Gene	Forward primer	Reverse primer
*Aif1*	GGATCAACAAGCAATTCCTCGA	CTGAGAAAGTCAGAGTAGCTGA
*B3gnt5*	CGTGGGGCAATGAGAACTAT	CCCAGCTGAACTGAAGAAGG
*Ccl2*	TTAAAAACCTGGATCGGAACCAA	GCATTAGCTTCAGATTTACGGGT
*Ccr2*	AGCACATGTGGTGAATCCAA	TGCCATCATAAAGGAGCCA
*Cd44*	ACCTTGGCCACCACTCCTAA	GCAGTAGGCTGAAGGGTTGT
*Cd44v6*	CTAATAGTACAGCAGAAGCAGCAGCTA	CCTGCCATCCGTTCTGAAA
*Csf1r*	AGGCAGGCTGGAATAATCTGACCT	CGTCACAGAACAGGACATCAGAGC
*Cx3cr1*	CAGCATCGACCGGTACCTT	GCTGCACTGTCCGGTTGTT
*Gbp2*	GGGGTCACTGTCTGACCACT	GGGAAACCTGGGATGAGATT
*Gfap*	AGAAAGGTTGAATCGCTGGA	CGGCGATAGTCGTTAGCTTC
*Itgam*	TGGCCTATACAAGCTTGGCTTT	AAAGGCCGTTACTGAGGTGG
*Psmb8*	CAGTCCTGAAGAGGCCTACG	CACTTTCACCCAACCGTCTT
*Ptx3*	AACAAGCTCTGTTGCCCATT	TCCCAAATGGAACATTGGAT
*Srgn*	GCAAGGTTATCCTGCTCGGA	TGGGAGGGCCGATGTTATTG
*Tmem119*	GTGTCTAACAGGCCCCAGAA	AGCCACGTGGTATCAAGGAG
*Tnf*	TGTGCTCAGAGCTTTCAACAA	CTTGATGGTGGTGCATGAGA
*Rpl19*	GAAGGTCAAAGGGAATGTGTTCA	CCTTGTCTGCCTTCAGCTTGT

### 2.11 Statistical analyses

Statistical analyses were performed in GraphPad Prism 6.01 (GraphPad Software Inc.). Survival curve analysis was performed by Log‐rank [Mantel Cox] Test. Image and gene expression analyses were performed by Student's *t*‐test (two groups) or ANOVA (four groups). Results are expressed as dot plots of individual animal observations with median values indicated (bar). CatWalkXT analysis was performed using two‐way ANOVA and expressed as group mean with 95% confidence interval. Values of *P* < .05 were accepted as significant.

## 3 RESULTS

### 3.1 *Csf1r* ^ΔFIRE^ mice rapidly succumb to prion disease in the absence of microglia

**FIGURE 1:**
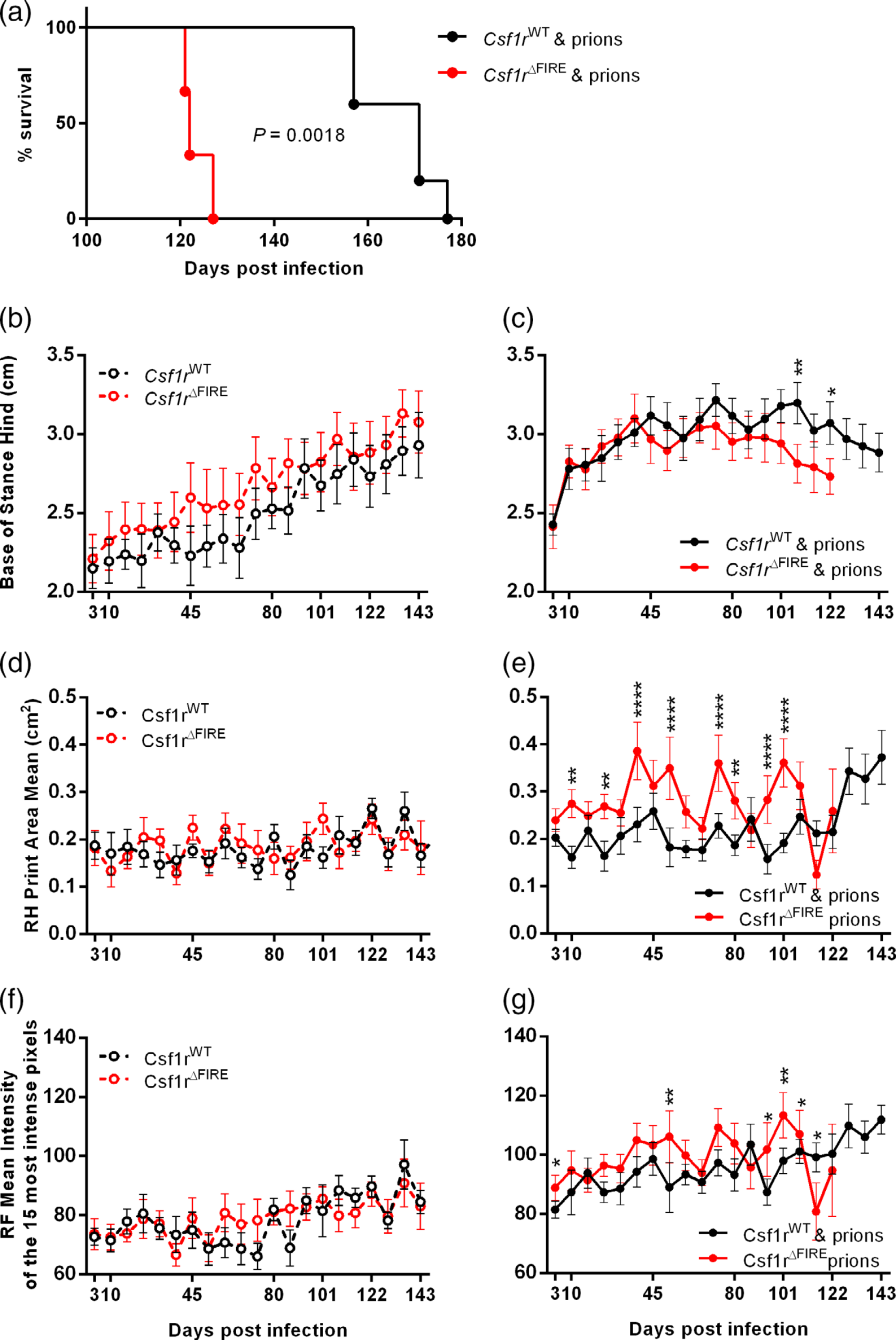
*Csf1r* ^ΔFIRE^ mice rapidly succumb to prion disease. (a) Survival curve following intracerebral injection of ME7 prions into *Csf1r* ^WT^ or *Csf1r* ^ΔFIRE^ mice (*N =* 5–6 mice/group). Log‐rank Mantel Cox test, *P* = .0018. (b) Catwalk XT automated gait analysis weekly assessment of hind base of stance in age‐matched uninfected *Csf1r* ^WT^ or *Csf1r* ^ΔFIRE^ mice. Points represent group mean and error bars 95% confidence interval. (c) Weekly assessment of hind base of stance in prion‐infected *Csf1r* ^WT^ or *Csf1r* ^ΔFIRE^ mice. (d) Weekly assessment of right hind (RH) paw print area in age‐matched uninfected mice. Two‐way ANOVA. (e) Weekly assessment of right hind (RH) paw print area in prion‐infected mice. (f) Weekly assessment of right front (RF) paw intensity in age‐matched uninfected mice. (g) Weekly assessment of right front (RF) paw intensity in prion‐infected mice. *P < .05; **P < .005; ****P < .0001; Two‐way ANOVA, Sidak's multiple comparisons test. Panels B‐G, *N =* 6–10 mice/group

To determine the role of microglia in prion disease, groups of homozygous microglia‐deficient *Csf1r* ^ΔFIRE^ transgenic mice and wild‐type (*Csf1r* ^WT^) littermate controls were injected intracerebrally (IC) with the ME7 strain of mouse adapted scrapie prions. As expected, all the *Csf1r* ^WT^ mice displayed clinical signs of prion disease from approximately 140 days after injection and succumbed to terminal disease with a mean survival time of 167 ± 5 days. In *Csf1r* ^ΔFIRE^ mice, clinical manifestations of prion disease were evident by 98 days after infection and progressed rapidly resulting in a mean survival time of 124 ± 2 days (Figure 1a).

### 3.2 Longitudinal gait analysis during prion infection

CNS prion disease in mice is associated with profound motor‐coordination disturbances (Heitzman & Corp, 1968). We therefore used longitudinal gait analysis to determine whether microglia‐deficiency affected the onset of motor disturbances during CNS prion disease (Figure 1b–g). Our analyses revealed no significant impact of the complete absence of microglia in the cerebellum of *Csf1r* ^ΔFIRE^ mice on motor function analyzed at any time point in the absence of the prion challenge (Figure 1b,d,f). As expected, various motor functions were rapidly impacted in prion disease. The base of stance (BOS, or distance between the hind paws) increased gradually with age in uninfected mice regardless of genotype (Figure 1b) but diverged by 10 days post infection (dpi) with prions and was maintained until 63 dpi (9 weeks) in *Csf1r* ^ΔFIRE^ mice and 108 dpi (15 weeks) in *Csf1r* ^WT^ mice (Figure 1c). At the onset of clinical signs of prion disease at 101 dpi *Csf1r* ^ΔFIRE^ mice were hyperactive and continued to perform Catwalk Gait analysis with ease until the terminal stage. In contrast, *Csf1r* ^WT^ mice at the onset of clinical symptoms at 143 dpi were severely ataxic and unable to cross the Catwalk within the time‐period required for data acquisition (Figure 1c).

Due to the potential effects of IC injection of prions into the right hemisphere on the contralateral paws, we analyzed the effects on footfall using only the unilateral right front and hind paws. In uninfected mice, hind paw area remained unchanged with no statistically significant differences between *Csf1r* ^WT^ and *Csf1r* ^ΔFIRE^ mice (Figure 1d). A significant increase in hind paw area was observed at 10 dpi in prion‐infected *Csf1r* ^ΔFIRE^ mice with a further large increase at 38 dpi. In contrast *Csf1r* ^WT^ mice did not experience a large increase in right hind paw area until 129 dpi, 3 weeks before commencement of clinical signs, these data are indicative of a more rapid response to prion infection in *Csf1r* ^ΔFIRE^ mice (Figure 1e).

In age‐matched uninfected mice, front paw intensity remained unchanged with no statistically significant differences between *Csf1r* ^WT^ and *Csf1r* ^ΔFIRE^ mice (Figure 1f). Concurrent with changes in footprint area, footfall intensity was increased in the prion‐infected mice (Figure 1g). Front footfall intensity increased significantly from 3 dpi in *Csf1r* ^WT^ mice and this increase was maintained almost throughout the duration of the prion infection until the terminal stage. In contrast, footfall intensity in the prion‐infected *Csf1r* ^ΔFIRE^ mice commenced at 10 dpi and was maintained until onset of clinical symptoms at 101 dpi (Figure 1g).

### 3.3 Detection of microgliosis in prion‐infected WT mice

**FIGURE 2:**
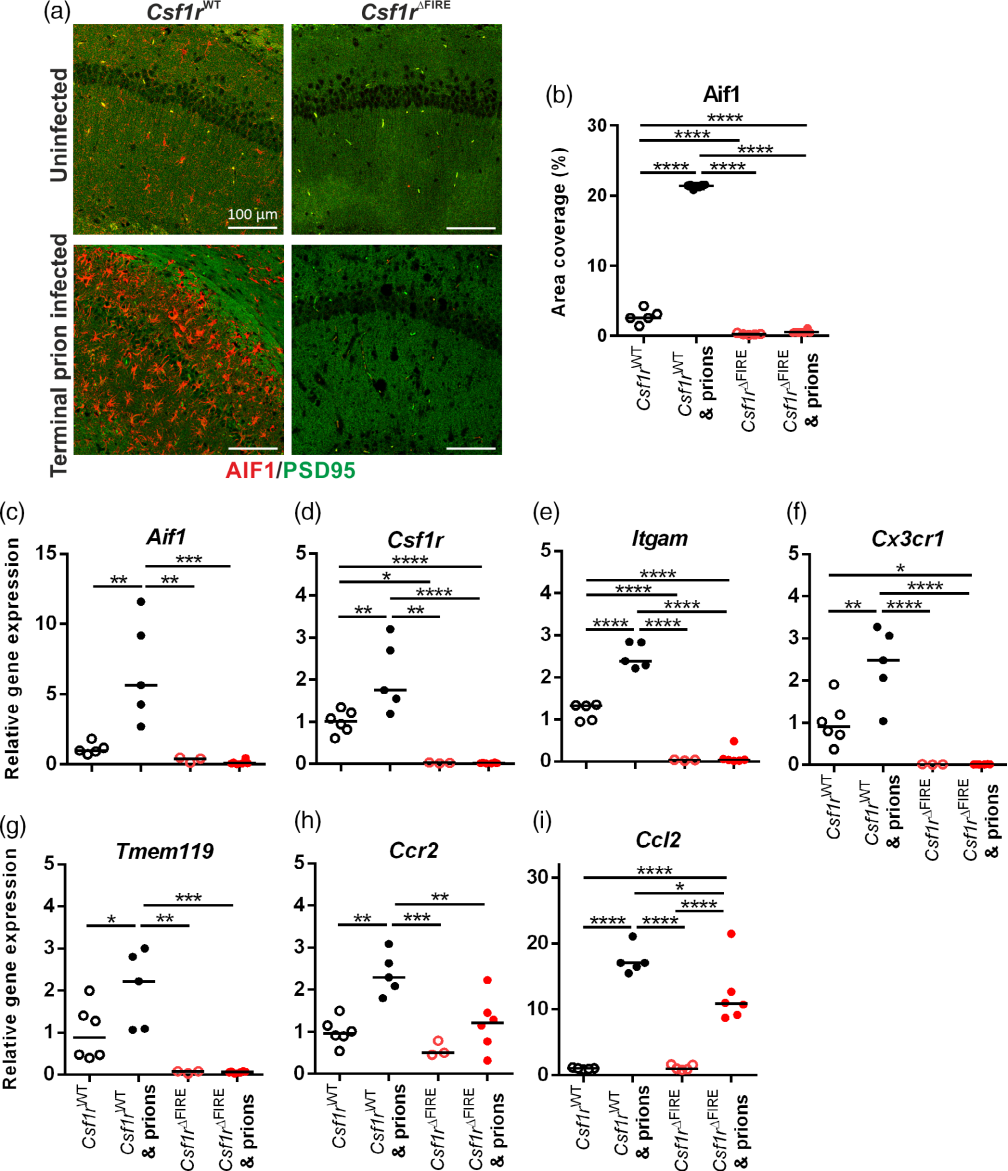
*Csf1r* ^ΔFIRE^ mice succumb to prion disease in the absence of microglia. (a) Immunohistochemical assessment of AIF1 (red) in hippocampus CA1 of terminal prion infected or age‐matched uninfected *Csf1r* ^WT^ or *Csf1r* ^ΔFIRE^ mice. Sections were counterstained to detect the post‐synaptic protein PSD95 (green). Scale bars = 100 μm. (b) AIF1 immunostaining quantitation expressed as % area coverage in hippocampus CA1. (c–i) RT‐qPCR of (c) *Aif1*, (d) *Csf1r*, (*e*) *Itgam*, (*f*) *Cx3cr1*, (*g*) *Tmem119*, (h) *Ccr2*, and (i) *Ccl2* mRNA in uninfected or terminal prion‐infected brains from *Csf1r* ^WT^ or *Csf1r* ^ΔFIRE^ mice. Points show individual mice. Horizontal bar = median. *P < .05; **P < .01; ****P < .0001; ANOVA. *N =* 5–6 mice/group

The brains of terminal prion‐infected *Csf1r* ^WT^ mice displayed abundant, activated microglia (allograft inhibitory factor‐1‐positive [AIF1^+^] cells), whereas these cells and other potential AIF1^+^ CNS‐infiltrating mononuclear phagocyte populations remained absent in uninfected and terminally‐affected *Csf1r* ^ΔFIRE^ mice, despite evidence of prion‐induced vacuolation and synaptic loss as revealed by co‐staining with the post‐synaptic protein PSD95 (Figure 2a,b) (Rojo et al., 2019). RT‐qPCR analysis confirmed that *Aif1* (Figure 2c) and *Csf1r* (Figure 2d) mRNA expression was significantly increased in the brains of terminally‐affected *Csf1r* ^WT^ mice when compared to age‐matched uninfected controls, but remained almost undetectable in the brains of *Csf1r* ^ΔFIRE^ mice even at the terminal stage of prion disease. Expression of other important microglia genes including *Itgam* (encoding CD11b; Figure 2e), *Cx3cr1* (Figure 2f) and *Tmem119* (Figure 2g) were also significantly increased in *Csf1r* ^WT^ mice, but absent in *Csf1r* ^ΔFIRE^ mice at the terminal stage of prion infection. Together, these data show that onset of CNS prion disease was accelerated in *Csf1r* ^ΔFIRE^ mice in the complete absence of microglia. The monocyte chemokine receptor *Ccr2* (Figure 2h) was significantly increased following prion infection in *Csf1r* ^WT^ mice, but not in *Csf1r* ^ΔFIRE^ mice, despite significantly increased expression of the monocyte chemoattractant *Ccl2* in brains of infected *Csf1r* ^WT^ mice and *Csf1r* ^ΔFIRE^ mice (Figure 2i). Notably, the *Csf1r* ^ΔFIRE^ mice are not monocyte‐deficient but their monocytes lack CSF1R expression (Rojo et al., 2019). The IHC and expression profiling indicates that the *Csf1r* ^ΔFIRE^ mutation also prevents monocyte recruitment into the injured brain. Why monocytes aren't recruited into the brains of *Csf1r* ^ΔFIRE^ mice is uncertain. Studies by Gómez‐Nicola and colleagues have similarly shown that CNS prion disease was not associated with significant monocytic recruitment in wild‐type mice, and the absence circulating monocytes in *Ccr2* ^−/−^ mice had little, if any, impact on the microgliosis or the progression of CNS disease (Gómez‐Nicola et al., 2014).

### 3.4 Unaltered neuronal loss but reduced prion accumulation in the brains of microglia‐deficient mice

**FIGURE 3:**
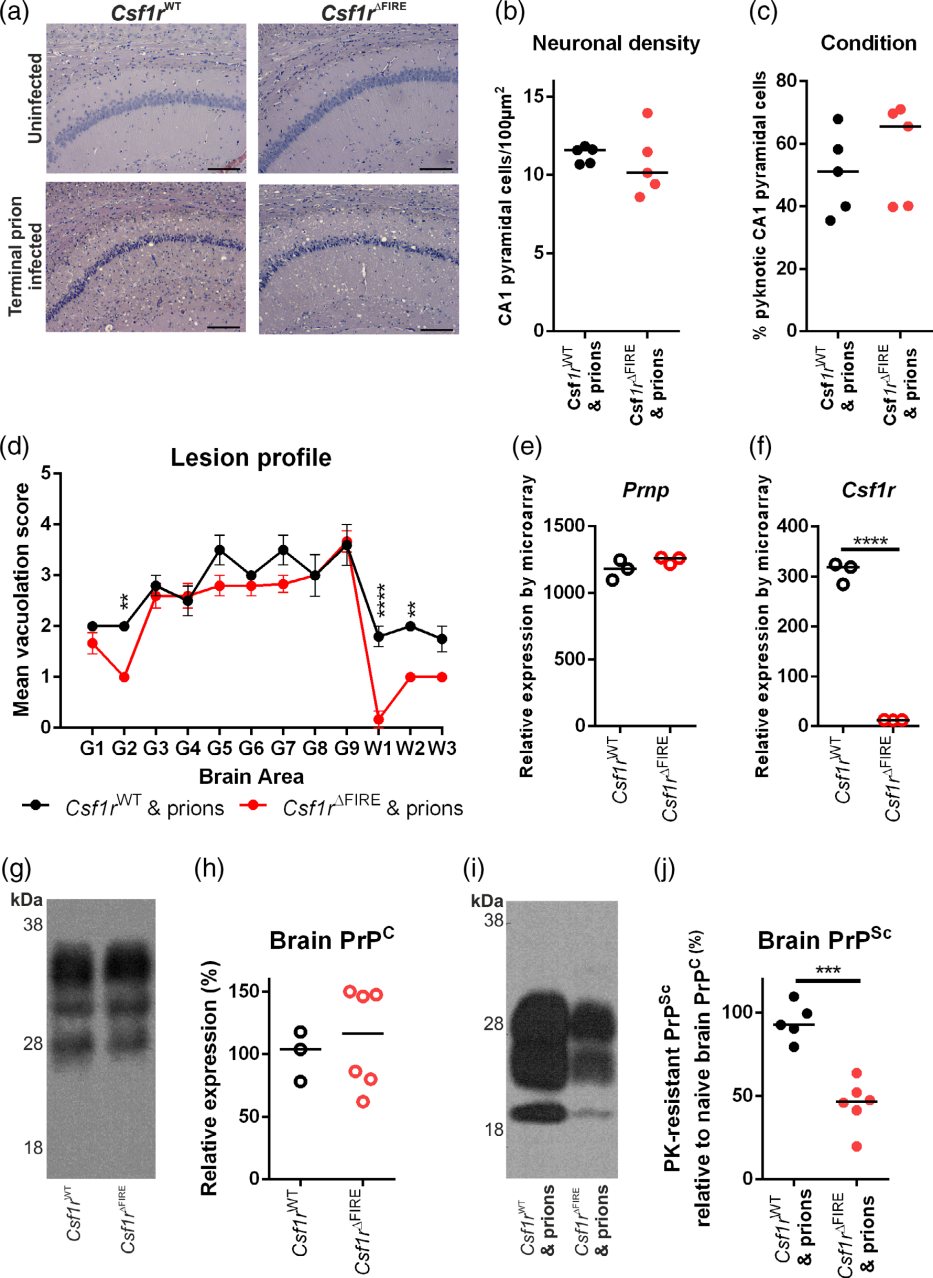
Microglia‐deficiency effects on prion‐specific vacuolation and prion accumulation. (a) Hematoxylin and eosin stained hippocampus CA1 of terminal prion infected or age‐matched uninfected *Csf1r* ^WT^ or *Csf1r* ^ΔFIRE^ mice. Scale bars = 200 μm. (b) Hippocampal CA1 pyramidal cell density in terminal prion infected *Csf1r* ^WT^ or *Csf1r* ^ΔFIRE^ mice. Student's *t*‐test. (c) Assessment of neuronal condition expressed as percentage of total neurons pyknotic in terminal prion infected *Csf1r* ^WT^ or *Csf1r* ^ΔFIRE^ mice. Student's *t*‐test. (d) Lesion profile analysis of prion‐infected brains. Points represent the mean vacuolation score, error bars = ± *SEM*. Two‐way ANOVA, Sidak's multiple comparisons test. **P < .005; ****P < .0001. (e) Microarray analysis of relative gene expression of *Prnp* in the brain. Student's *t*‐test. (f) Microarray analysis of relative gene expression of *Csf1* in the brain. Student's *t*‐test, ****P < .0001. (g) Western blot analysis of uninfected *Csf1r* ^WT^ and *Cs1fr* ^ΔFIRE^ mouse brain, probed with anti‐PrP antibody clone BH1. Relative protein sizes indicated in kilodaltons (kDa). (h) Quantitation of relative brain PrP^C^ expression in the brains of uninfected *Csf1r* ^WT^ and *Cs1fr* ^ΔFIRE^ mice. Student's *t*‐test. (i) Western blot analysis of terminal prion‐infected *Csf1r* ^WT^ and *Cs1fr* ^ΔFIRE^ mouse brain, probed with anti‐PrP antibody clone BH1. Relative protein sizes indicated in kilodaltons (kDa). (j) Quantitation of relative PrP^Sc^ accumulation in the brains of terminal prion‐infected *Csf1r* ^WT^ and *Cs1fr* ^ΔFIRE^ mice. Students *t*‐test. ***P < .001. Panels a–d, *N =* 5–6 mice/group. Panels e and f, 3 mice/group. Panels g–j, *N =* 3–6 mice/group. Panels b, c, e, f, h, and j. points show individual mice, horizontal bar = median.

Assessment of hippocampal CA1 pyramidal cells in hematoxylin and eosin stained brain sections (Figure 3a) revealed no difference in neuronal density or the frequency of pyknotic (apoptotic) neuronal nuclei between terminal prion‐infected *Csf1r* ^WT^ and *Csf1r* ^ΔFIRE^ mice despite the difference in time of onset of pathology (Figure 3b,c, respectively). The prion‐specific vacuolation was also comparable in most brain areas of terminal prion‐infected *Csf1r* ^WT^ and *Csf1r* ^ΔFIRE^ mice, except for a significant reduction of vacuolation in the cerebellar cortex (G2), inferior and middle cerebellar peduncles (W1) and decussation of superior cerebellar peduncles (W2) of brains from prion‐infected *Csf1r* ^ΔFIRE^ mice (Figure 3d). This suggested the pathological impact of prion infection upon the cerebellum was reduced in the *Csf1r* ^ΔFIRE^ mice at the terminal stage of prion disease.

The relative expression level of PrP^C^ can directly influence prion disease duration (Manson et al., 1994). Previous expression profiling of the cortex of *Csf1r* ^ΔFIRE^ compared to *Csf1r* ^WT^ mice revealed no impacts on expression of *Prnp* mRNA (which encodes PrP^C^) or any other neuron‐associated transcripts (Rojo et al., 2019). Expression of *Prnp* mRNA in the hippocampus in published mRNA microarray data GEO dataset GSE108207 (Rojo et al., 2019) (Figure 3e) was similar in each mouse strain, despite loss of *Csf1r* expression (Figure 3f). Whole brain PrP^C^ protein expression (Figure 3g,h) was also similar between naïve *Csf1r* ^ΔFIRE^ mice and *Csf1r* ^WT^ mice. Partial‐deficiency or temporary ablation of microglia during CNS prion infection was reported to accelerate the accumulation of prion‐disease‐specific PrP^Sc^ in the brain (Carroll et al., 2018; Zhu et al., 2016). By contrast, PrP^Sc^ accumulation was reduced in the brains of terminally prion‐infected *Csf1r* ^ΔFIRE^ compared to *Csf1r* ^WT^ mice (Figure 3i,j).

### 3.5 Altered neuropathology in the absence of microglia during CNS prion disease

**FIGURE 4:**
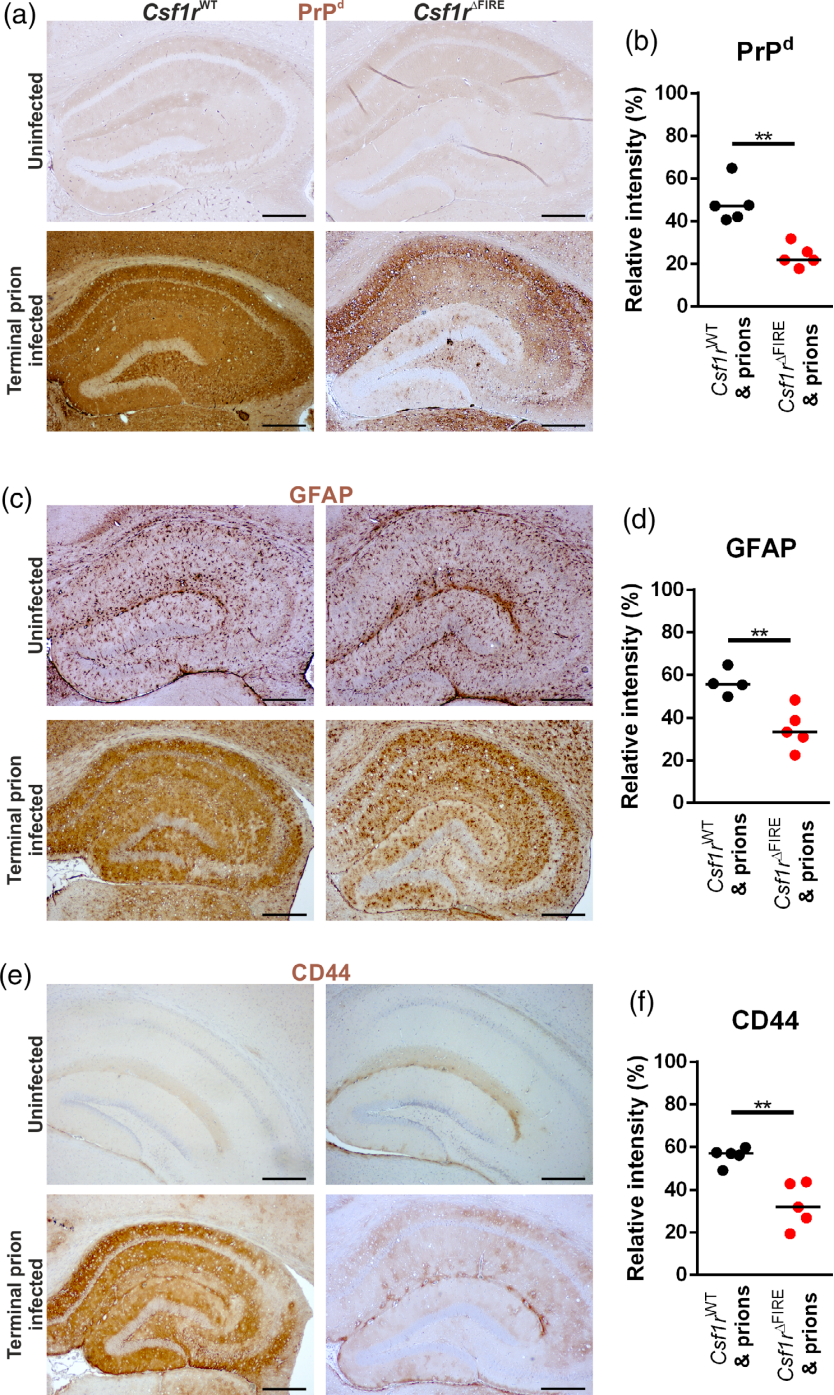
Microglial deficiency reduces terminal neuropathology. (a, c, e) Immunohistochemical assessment of (a) PrP^d^ accumulation, (c) GFAP expression and (e) CD44 expression (brown) in the hippocampus of terminal prion infected or age‐matched uninfected *Csf1r* ^WT^ and *Cs1fr* ^ΔFIRE^ mice. DAB (brown) immunostaining lightly counterstained with hematoxylin (blue). Scale bars = 500 μm. (b) PrP^d^ immunostaining quantified by relative intensity. (d) GFAP immunostaining quantified by relative intensity. (f) CD44 immunostaining quantified by relative intensity. Points show individual mice. Horizontal bar = median. Student's *t*‐test, **P < .005. *N =* 4–5 mice/group

Consistent with data presented in Figure 3i,j, immunostaining for prion disease‐associated PrP (PrP^d^) in the brains of *Csf1r* ^ΔFIRE^ mice at the terminal stage was approximately 50% of the intensity detected in *Csf1r* ^WT^ mice (Figure 4a,b). Since the accumulation of PrP^Sc^ within the brain increases as the infection proceeds (Tatzelt et al., 1999), this finding is most likely a consequence of their significantly shortened survival times, and implies that microglia deficiency produces hyper‐sensitivity to the accumulation of PrP^Sc^.

CNS prion disease is accompanied by extensive reactive astrocytosis characterized by high levels of expression of glial fibrillary acidic protein (GFAP), CD44 and the CD44v6 alternative splice variant (Bradford et al., 2019). Microglia and microglial‐derived factors have been shown to induce reactive astrocytosis in a range of neurodegenerative conditions (Kunyu et al., 2019; Liddelow et al., 2017; Vainchtein & Molofsky, 2020). Despite the absence of microglia, reactive astrocytes expressing high levels of GFAP (Figure 4c,d) and CD44 (Figure 4e,f) were increased in the brains of prion‐infected *Csf1r* ^ΔFIRE^ mice but the level of GFAP^+^ and CD44^+^ immunostaining was lower than in infected *Csf1r* ^WT^ mice. As astrocyte activation also increases temporally during CNS prion infection (Bradford et al., 2019; Hwang et al., 2009), this again is most likely a consequence of the *Csf1r* ^ΔFIRE^ mice succumbing to terminal prion disease significantly earlier than infected *Csf1r* ^WT^ mice. In summary, these data reveal that although CNS prion disease duration is shorter in microglia‐deficient *Csf1r* ^ΔFIRE^ mice, this is not accompanied by increased neuronal vacuolation, prion accumulation, or upregulation of GFAP or CD44 at the terminal stage, when compared to infected *Csf1r* ^WT^ mice.

### 3.6 Absence of induction of neurotoxic “A1” or neuroprotective “A2” reactive astrocyte‐associated genes in the brains of prion‐infected microglia‐deficient mice

**FIGURE 5:**
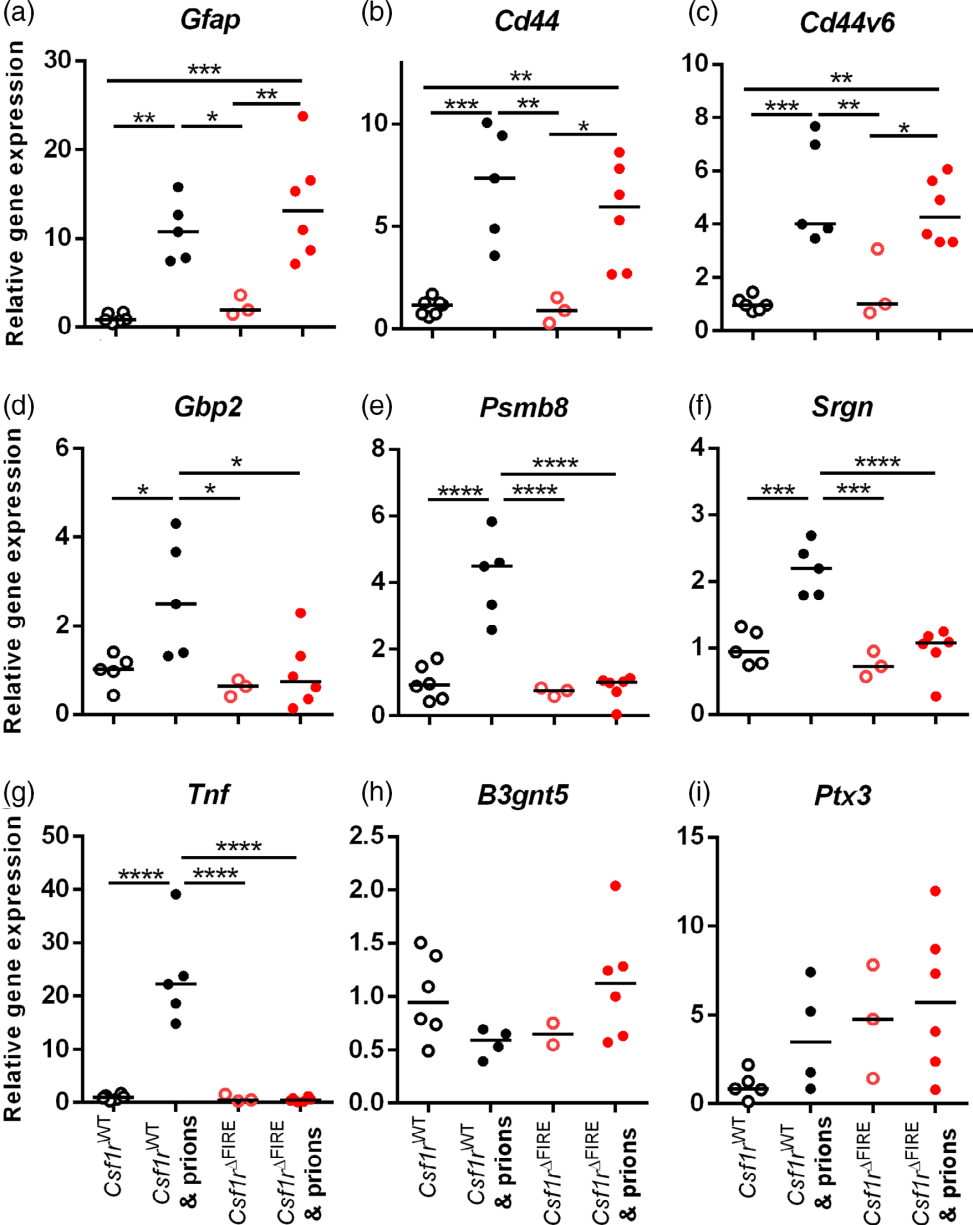
Microglia‐deficiency alters astrocyte response to prions. RT‐qPCR analysis of (a) *Gfap*, (b) *Cd44*, (c) *Cd44v6*, (d) *Gbp2*, (e) *Psmb8*, (f) *Srgn*, (g) *Tnf*, (h) *B3gnt5*, and (i) *Ptx3* mRNA in the brains of terminal prion infected or age‐matched uninfected *Csf1r* ^WT^ or *Csf1r* ^ΔFIRE^ mice. Points show individual mice. Horizontal bar = median. *P < .05; **P < .005; ***P < .001; ****P < .0001; ANOVA. *N =* 3–6 mice/group

Reactive astrocytes may be classified into distinct functional subclasses; an A1 subclass with neurotoxic activity and A2 astrocytes considered neurotrophic (Liddelow et al., 2017). Microglia‐derived factors have been implicated in the induction of pan‐ and A1‐reactive astrocyte‐associated genes (Liddelow et al., 2017). Consistent with the immunohistochemistry data presented in Figure 4, high levels of mRNA encoding the pan‐reactive astrocyte‐associated genes *Gfap*, *Cd44*, and *Cd44v6* were detected in the brains of prion‐infected *Csf1r* ^WT^ mice (Figure 5a–c, respectively). The LPS‐mediated induction of expression of pan‐reactive astrocyte‐associated genes including *Gfap* and *Cd44* was reported to be blocked in microglia‐deficient *Csf1r* ^−/−^ mice (Liddelow et al., 2017). However, because of the limited viability of *Csf1r* ^−/−^ mice, these studies were performed at postnatal day 8, and these mice are also deficient in peripheral macrophage populations. In the *Csf1r* ^ΔFIRE^ mice, the expression of *Gfap*, *Cd44*, and *Cd44v6* was upregulated in response to prion infection despite the complete absence of microglia. These data demonstrate CNS prion‐induced reactive astrocyte activation is not dependent on the presence of microglia.

At the terminal stage of prion disease, the reactive astrocytes display a dysregulated transcriptional signature including expression of both A1 and A2 astrocyte‐associated genes (Donaldson et al., 2020; Hartmann et al., 2019). The expression of the neurotoxic A1 astrocyte‐associated genes *Gbp2, Psmb8*, and *Srgn* was upregulated in the brains of terminal prion‐infected *Csf1r* ^WT^ mice, but absent in *Csf1r* ^ΔFIRE^ mice (Figure 5d–f). Microglia‐derived cytokines including tumor necrosis factor (TNFα) are important inducers of neurotoxic A1 reactive astrocyte activation. Indeed, *Tnf* was elevated in the brains of prion‐infected *Csf1r* ^WT^ mice but absent in *Csf1r* ^ΔFIRE^ mice, coincident with the lack of induction of A1 reactive astrocyte‐associated gene expression. Consistent with previous data from the brains of mice infected with ME7 scrapie prions (Donaldson et al., 2020), neuroprotective A2 astrocyte‐associated genes (*B3gnt5* and *Ptx3*) were not induced in the brains of infected *Csf1r* ^WT^ or *Csf1r* ^ΔFIRE^ mice (Figure 5h,i). Together these data show that CNS prion disease in microglia‐deficient *Csf1r* ^ΔFIRE^ mice is accompanied by dysregulated reactive astrocytosis that lacks evidence of a typical neurotoxic A1 or neuroprotective A2 transcriptional profile.

### 3.7 *Csf1r* ^ΔFIRE^ mice display accelerated onset of vacuolation but unaltered kinetics of prion accumulation

**FIGURE 6:**
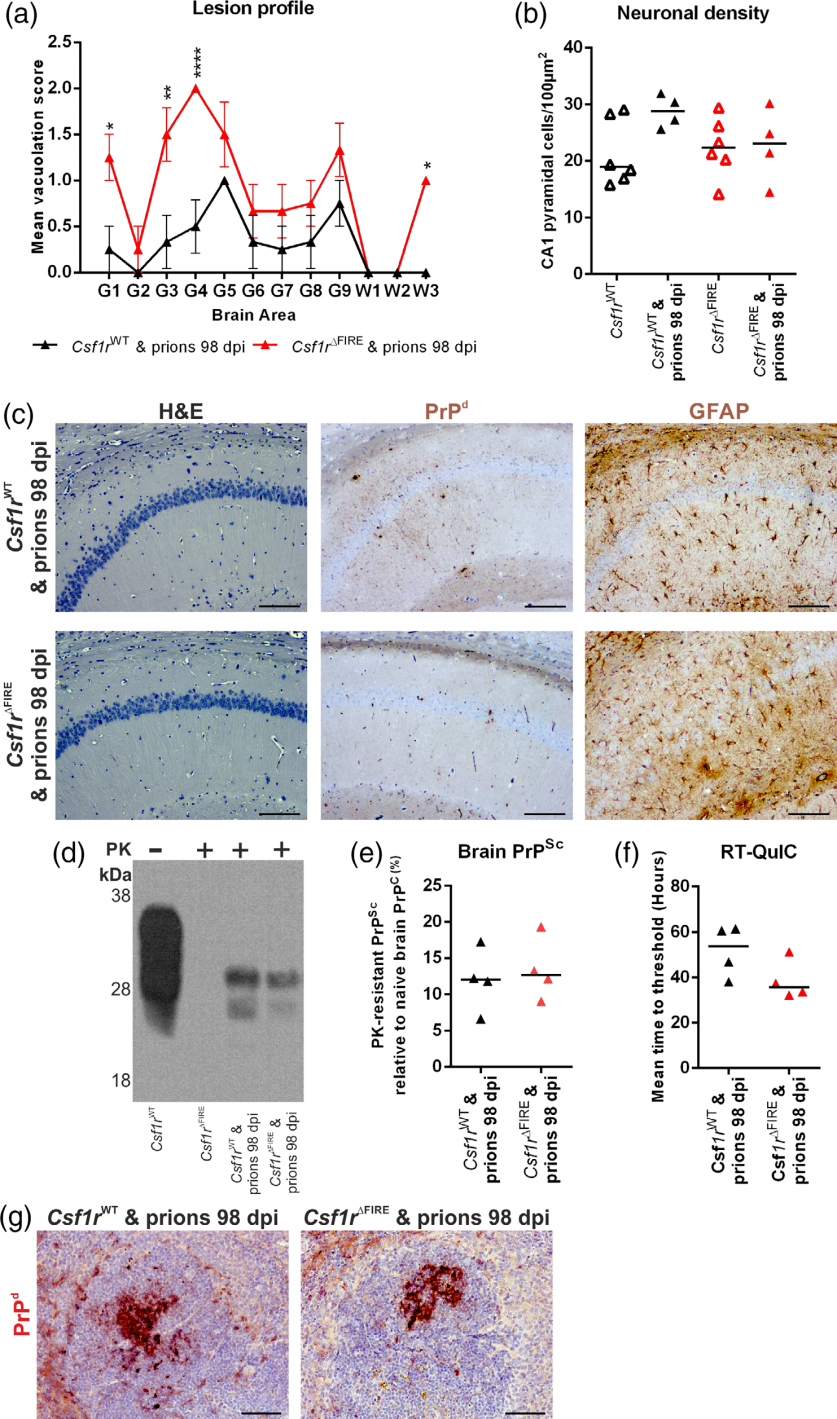
Microglial deficiency accelerates prion vacuolation but not brain or peripheral prion accumulation. (a) Lesion profile analysis of prion‐infected brains at 98 dpi (*N =* 4 mice/group). Points represent the mean vacuolation score, error bars = *SEM*. *P < .05; **P < .01; ****P < .0001; two‐way ANOVA, Sidak's multiple comparisons test. (b) Hippocampal CA1 pyramidal neuron density was assessed in 98 dpi prion‐infected mice and age‐matched uninfected (*N =* 4–6 mice/group). Points show individual mice, bar = median. Not significantly different, ANOVA. (c) Hematoxylin and eosin (H&E) stained sections used for vacuolation and neuronal density analyses. Immunohistochemical analysis of PrP^d^ accumulation and GFAP expression in 98 dpi prion‐infected *Csf1r* ^WT^ and *Csf1r* ^ΔFIRE^ hippocampus CA1. Scale bars = 200 μm. (d) Western blot analysis as indicated to determine the relative amount of PrP^Sc^ accumulation in the brains of mice from each group at 98 dpi with prions. (e) Quantitation of PrP^Sc^ levels in brains of 98 dpi prion‐infected *Csf1r* ^WT^ and *Csf1r* ^ΔFIRE^ mice. Points show individual mice, bar = median. Not significantly different, Student's *t*‐test. (F) Relative prion seeding activities in brains at 98 dpi with prions were quantified in vitro by RT‐QuIC expressed as mean time to threshold. Points show individual mice, bar = median. Not significantly different, Student's *t*‐test. (g) Immunohistochemical analysis of PrP^d^ accumulation in spleens of prion‐infected *Csf1r* ^WT^ and *Csf1r* ^ΔFIRE^ mice at 98 dpi. PrP^d^ immunostaining (red) counterstained with hematoxylin (blue). Scale bar = 100 μm. Panels c–g, *N =* 4 mice/group

To determine how disease progression was affected by the absence of microglia, brains were collected from groups of *Csf1r* ^WT^ and *Csf1r* ^ΔFIRE^ mice at 98 dpi prior to the histopathological detection of neuronal loss. Prion‐specific vacuolation was already more severe in prion‐infected *Csf1r* ^ΔFIRE^ mice in multiple brain regions, including dorsal medulla, superior colliculus, hypothalamus and cerebellar peduncles (Figure 6a; vacuolation scoring areas G1, G3, G4, and W3). However, within the hippocampus little evidence of prion‐specific vacuolation (Figure 6a, vacuolation scoring area G6; Figure 6c upper panels) or neuronal loss (Figure 6b) was observed in brains from each group at this time. The early “synaptic” patterned PrP^d^ deposition and mild reactive astrocytosis also presented to a similar extent in the hippocampus of infected *Csf1r* ^ΔFIRE^ and *Csf1r* ^WT^ mice at this time point (Figure 6c, middle and lower panels, respectively).

The levels of PrP^Sc^ in the brains of *Csf1r* ^ΔFIRE^ or *Csf1r* ^WT^ mice at 98 dpi were indistinguishable (Figure 6d,e). In parallel, the highly sensitive real‐time quaking‐induced conversion (RT‐QuIC) assay was used to quantify the relative prion seeding activity present within the brains of each group (Atarashi et al., 2011). Consistent with data presented in Figure 6c–e, the relative levels of prion seeding activity were also similar in the brains of infected *Csf1r* ^ΔFIRE^ mice and *Csf1r* ^WT^ mice (Figure 6f).

After IC injection, some of the infectious prions from the inoculum spread to the spleen via the bloodstream where they accumulate on stromal follicular dendritic cells (FDC) (Brown et al., 1999). Following accumulation within the spleen and other secondary lymphoid organs, the prions can subsequently spread back to the brain (Brown et al., 2012; Brown & Mabbott, 2014). In the absence of peripheral macrophages, the accumulation of prions in secondary lymphoid tissues is enhanced (Beringue et al., 2000; Maignien et al., 2005). Since certain peripheral macrophages will also have been ablated in the previous studies (Carroll et al., 2018; Lei et al., 2020; Zhu et al., 2016) it is plausible that this may have increased the burden of prions in the spleen and other secondary lymphoid organs, and by doing so, enhanced their rate of spread to the brain. However, such an effect was unlikely to responsible for the accelerated prion disease in *Csf1r* ^ΔFIRE^ mice, as a similar abundance of prion‐specific PrP^d^ was detected on FDC in the spleens of *Csf1r* ^ΔFIRE^ mice and *Csf1r* ^WT^ mice (Figure 6g). This is consistent with the demonstration that spleen macrophage populations are not affected in *Csf1r* ^ΔFIRE^ mice (Rojo et al., 2019).

### 3.8 Accelerated onset of reactive astrocyte activation in the absence of microglia

**FIGURE 7:**
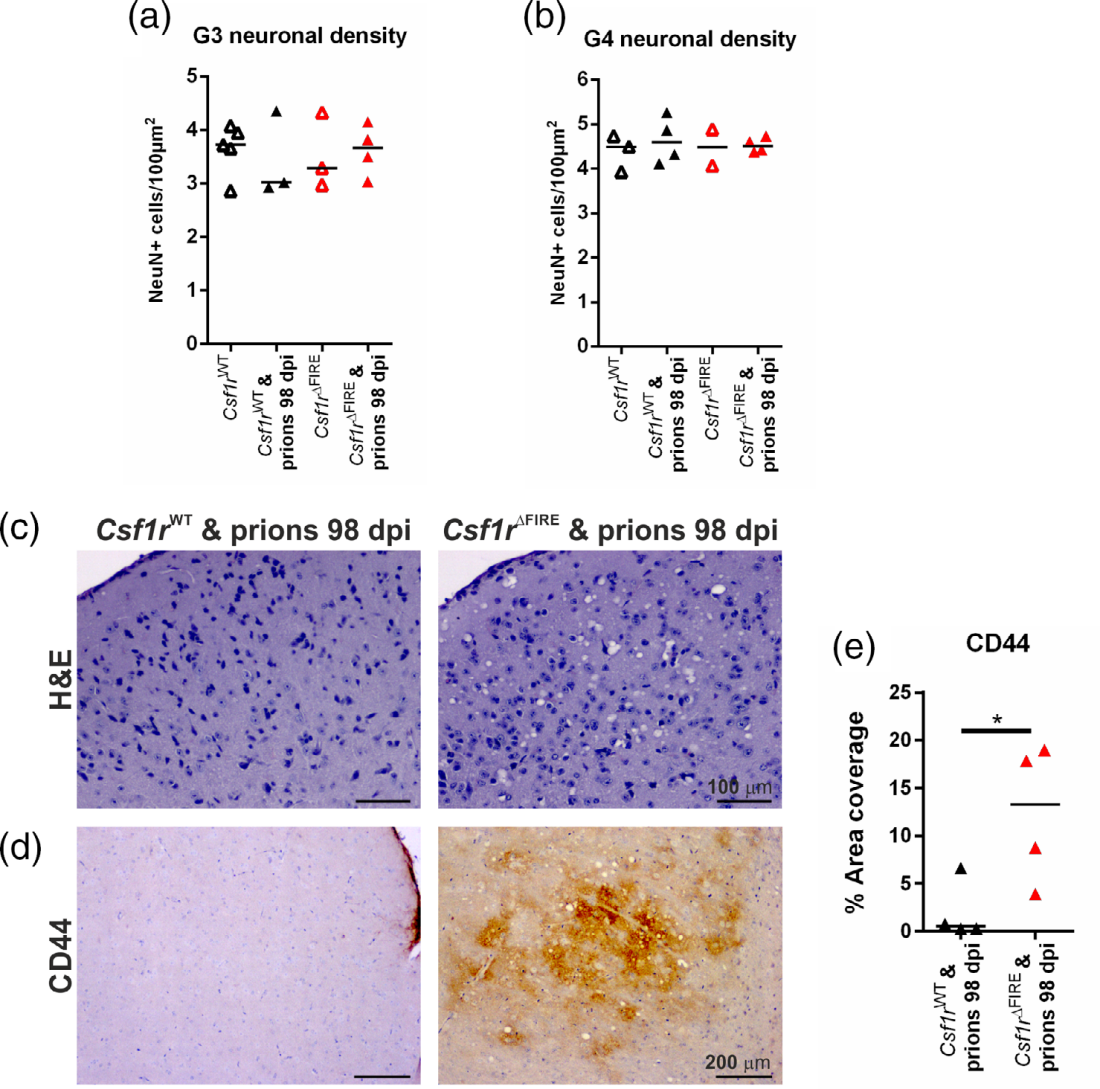
Accelerated astrocyte activation in the absence of microglia. (a) Superior colliculus (G3) and (b) hypothalamus (G4) neuronal density was assessed via quantitation of the density of NeuN^+^ cells in 98 dpi prion‐infected or age‐matched uninfected *Csf1r* ^WT^ and *Csf1r* ^ΔFIRE^ mice. Not significantly different, ANOVA. *N =* 2–5 mice/group. (c) Hematoxylin and eosin (H&E) stained superior colliculus in 98 dpi prion‐infected *Csf1r* ^WT^ and *Csf1r* ^ΔFIRE^ mice. Scale bars = 100 μm. (d) Immunohistochemical assessment of CD44 expression in 98 dpi prion‐infected *Csf1r* ^WT^ and *Cs1fr* ^ΔFIRE^ superior colliculus. Scale bars = 200 μm. (e) Quantitation of CD44% area coverage in superior colliculus. Points show individual mice. Horizontal bar = median. *P < .05, Student's *t* test. *N =* 4 mice/group

**FIGURE 8:**
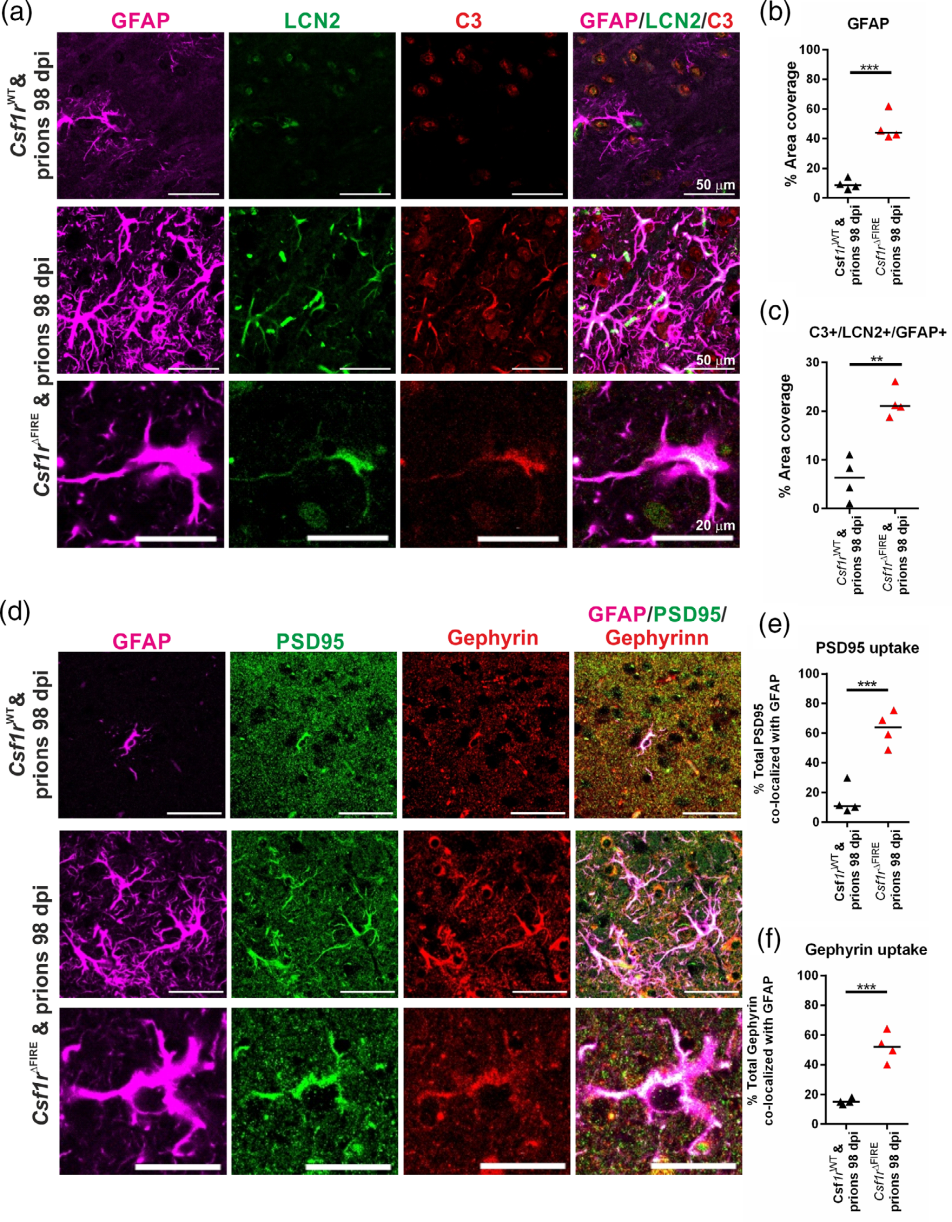
Increased astrocyte synaptic phagocytosis in the absence of microglia (a) Immunofluorescent assessment of GFAP (violet), lipocalin2 (LCN2, green) and complement component C3 (red) in 98 dpi prion‐infected *Csf1r* ^WT^ and *Cs1fr* ^ΔFIRE^ superior colliculus. Scale bars = 50 or 20 μm as indicated. (b) Quantitation of GFAP % area coverage in superior colliculus. (c) Quantitation of C3^+^/LCN2^+^/GFAP^+^ astrocytes. (d) Immunofluorescent assessment of GFAP (violet), and the post‐synaptic proteins PSD95 (green) and gephyrin (red) in 98 dpi prion‐infected *Csf1r* ^WT^ and *Cs1fr* ^ΔFIRE^ superior colliculus. Scale bars = 50 or 20 μm as indicated. (E) quantitation of PSD95 uptake by astrocytes expressed as % of total PSD95 colocalized with GFAP. (F) Quantitation of gephyrin uptake by astrocytes expressed as % of total gephryin colocalized with GFAP. Points show individual mice. Horizontal bar = median. **P < .01; ***P < .001, Student's T test. *N =* 4 mice/group

The increased prion‐specific vacuolation in multiple brain regions by 98 dpi (Figure 6a), for example within the superior colliculus and hypothalamus (vacuolation scoring areas G3 and G4, respectively) in the *Csf1r* ^ΔFIRE^ mice, was not accompanied by evidence of loss of neuronal nuclear antigen NeuN^+^ neurons within these regions in either *Csf1r* ^ΔFIRE^ or *Csf1r* ^WT^ mice at this time (Figure 7a,b). Instead, the increased vacuolation (Figure 7c) in the intermediate gray layer (motor associated area) of the superior colliculus of prion‐infected *Csf1r* ^ΔFIRE^ mice compared to *Csf1r* ^WT^ mice at 98 dpi was accompanied by increased expression of the pan‐astrocytic activation marker CD44 (Figure 7d,e) (Bradford et al., 2019) and increased frequency of GFAP^+^ morphologically reactive astrocytes (Figure 8a,b).

The innate immune proteins complement component C3 and neutrophil gelatinase‐associated lipocalin/lipocalin‐2 (NGAL/LCN2) have previously been shown to be upregulated by neurotoxic astrocytes in response to prion infection (Hartmann et al., 2019; Kushwaha et al., 2021; Smith et al., 2020). We observed a greater abundance of C3^+^/LCN2^+^/GFAP^+^ morphologically reactive astrocytes within the superior colliculus in *Csf1r* ^ΔFIRE^ compared to *Csf1r* ^WT^ mice at 98 dpi (Figure 8a,c).

Astrocytes in the steady state prune synapses to help maintain neural circuitry (Chung et al., 2013). However, abnormal astrocyte synaptic engulfment has been implicated in the pathogenesis of some neurodegenerative disorders (reviewed in Lee & Chung, 2019), and synaptic alterations are considered to contribute to the early behavioral changes observed during CNS prion disease (Cunningham et al., 2003). We therefore assessed the localization of the post‐synaptic proteins gephyrin and post‐synaptic density protein 95 (PSD95) in relation to GFAP^+^ astrocytes (Figure 8d). The co‐localization of both post‐synaptic marker proteins in punctate inclusions within GFAP^+^ morphologically reactive astrocytes was increased in the superior colliculus of *Csf1r* ^ΔFIRE^ compared to *Csf1r* ^WT^ mice at 98 dpi (Figure 8d). Morphometric analyses suggested over half of the total amount of these synaptic proteins detected in *Csf1r* ^ΔFIRE^ mice were within astrocytes (Figure 8e,f). Furthermore, additional analyses suggested that the preferential co‐localization of PSD95 and gephyrin within the GFAP+ reactive astrocytes of prion‐infected *Csf1r* ^ΔFIRE^ mice was highly significant compared to the null hypothesis that the immunostaining was randomly distributed (PSD95/GFAP, *P* < .0002; Gephyrin/GFAP, *P* < 7 × 10^−5^). Together, these data reveal a statistically significant increase in synaptic engulfment by reactive astrocytes in the brains of prion‐infected *Csf1r* ^ΔFIRE^ mice compared to *Csf1r* ^WT^ mice at 98 dpi within this region.

### 3.9 Accelerated onset of unfolded protein response in the absence of microglia

Accumulation of misfolded PrP^Sc^ in the brain triggers the unfolded protein response in reactive astrocytes (Smith et al., 2020). Specifically, phosphorylation of protein kinase‐like endoplasmic reticulum kinase (PERK) causes the transient shutdown of protein synthesis via phosphorylation of eukaryotic translation initiation factor 2A (eIF2α). Inhibition of PERK‐eIF2α signaling in astrocytes alleviated prion‐induced neurodegeneration (Smith et al., 2020).

**FIGURE 9:**
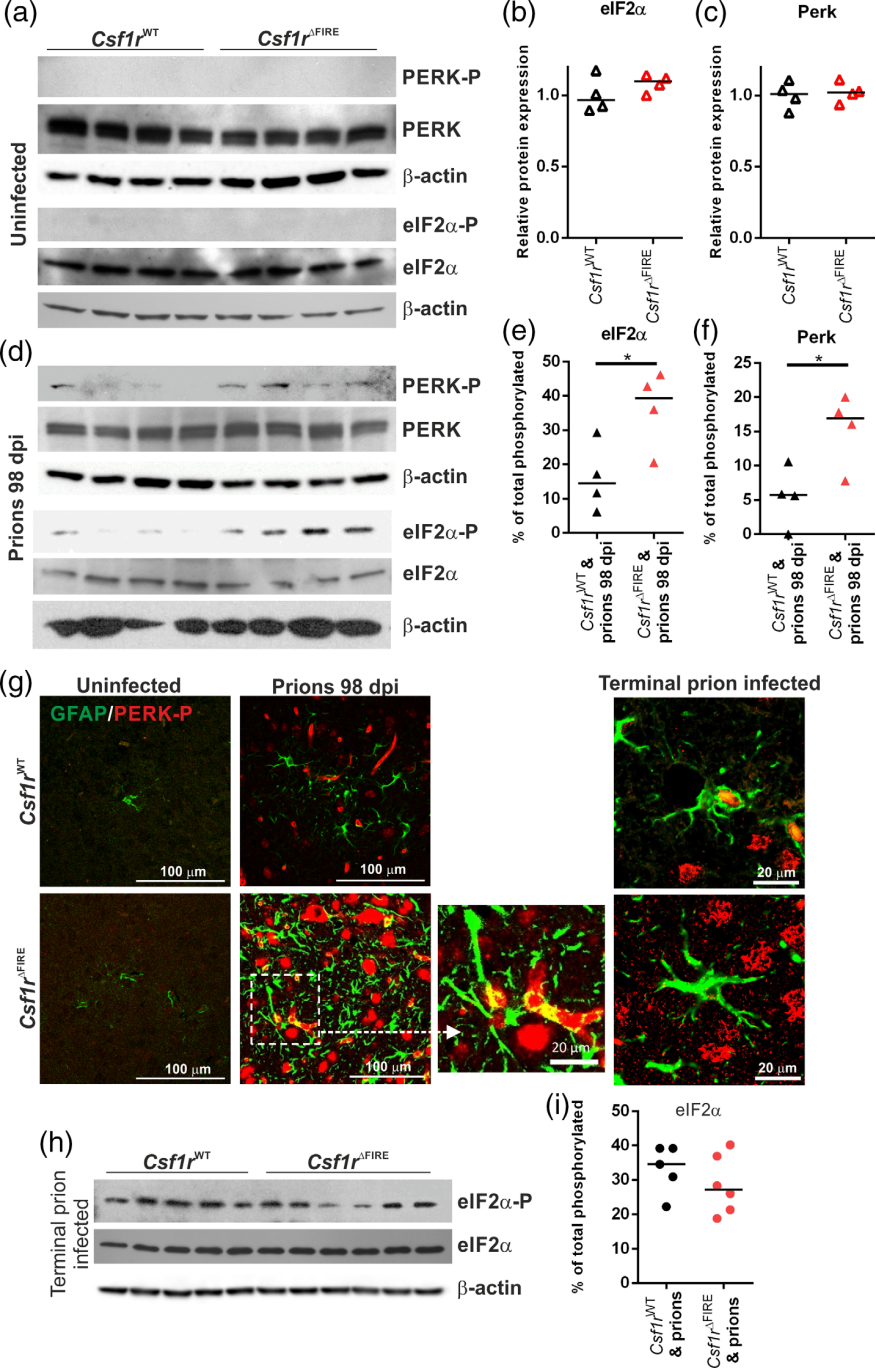
Increased unfolded protein response pathway is associated with earlier astrocyte activation. (a) Western blot analyses of age‐matched uninfected *Csf1r* ^WT^ and *Cs1fr* ^ΔFIRE^ mouse brains for unfolded protein response components as indicated, β‐Actin displayed as a loading control. (b) Quantitation of relative expression levels of eIF2α in uninfected *Csf1r* ^WT^ and *Cs1fr* ^ΔFIRE^ mouse brains. Not significantly different, Student's *t*‐test. (c) Quantitation of relative expression levels of PERK uninfected *Csf1r* ^WT^ and *Cs1fr* ^ΔFIRE^ mouse brain. Not significantly different, Student's *t*‐test. (d) Western blot analysis of 98 dpi prion‐infected *Csf1r* ^WT^ and *Cs1fr* ^ΔFIRE^ mouse brain for unfolded protein response components as indicated. (e) Quantitation of the percentage of total phosphorylated eIF2α in 98 dpi prion‐infected *Csf1r* ^WT^ and *Cs1fr* ^ΔFIRE^ mouse brain. *P < .05, Student's *t*‐test. (f) Quantitation of the percentage of total phosphorylated PERK in 98 dpi prion‐infected *Csf1r* ^WT^ and *Cs1fr* ^ΔFIRE^ mouse brain. *P < .05, Student's *t*‐test. (g) Immunohistochemical analysis of phosphorylated PERK (PERK‐P; red) and GFAP (green) in 98 dpi prion infected, terminal prion infected and age‐matched uninfected *Csf1r* ^WT^ and *Cs1fr* ^ΔFIRE^ superior colliculus (G3). Scale bars = 100 μm or 20 μm as indicated. (h) Western blot analysis of terminal prion‐infected brain homogenates probed for unfolded protein response components as indicated, β‐Actin displayed as a loading control. (i) Quantitation of the percentage of total phosphorylated eIF2α in terminal prion‐infected *Csf1r* ^WT^ and *Cs1fr* ^ΔFIRE^ mouse brains. Not significantly different, Student's *t*‐test. Points show individual mice. Panels A‐G, *N =* 4 mice/group. Horizontal bar = median. Panels H&I, *N =* 5–6 mice/group

Levels of PERK and eIF2α expression were assessed in brains of age‐matched uninfected mice and revealed no difference in uninfected mice *Csf1r* ^WT^ and *Csf1r* ^ΔFIRE^ mice (Figure 9a–c). Similarly, in uninfected mice we were unable to detect phosphorylated PERK and eIF2α (Figure 9a). However, the levels of phosphorylated PERK and eIF2α were statistically significantly increased in the brains of infected *Csf1r* ^ΔFIRE^ mice when compared to infected *Csf1r* ^WT^ mice at 98 dpi (Figure 9d–f). Immunohistochemical analysis also revealed earlier expression of phosphorylated PERK expression in GFAP^+^ reactive astrocytes and neurons in infected *Csf1r* ^ΔFIRE^ mice, particularly within the superior colliculus (Figure 9g), coincident with the increased vacuolation and reactive astrocytosis in this region at 98 dpi (Figure 7c). Conversely, little if any, phosphorylated PERK expression in GFAP^+^ reactive astrocytes was detected in infected *Csf1r* ^WT^ mice at 98 dpi (Figure 6g).

However, by the terminal stage of prion infection similar levels of phosphorylated eIF2α were detected in the brains of each mouse group despite the *Csf1r* ^ΔFIRE^ mice succumbing to clinical prion disease earlier (Figure 9h,i). Thus, these data suggest that the earlier astrocyte activation and neuronal vacuolation in the prion‐infected *Csf1r* ^ΔFIRE^ mice was accompanied by an increased unfolded protein response.

## 4 DISCUSSION

In this study, we investigated prion neuropathogenesis in microglia‐deficient *Csf1r* ^ΔFIRE^ mice. Spongiform vacuolation and neuronal loss at the terminal stage were indistinguishable in *Csf1r* ^WT^ and *Csf1r* ^ΔFIRE^ mice and the onset of pathology was not correlated with the accumulation of misfolded prions, which are in any case not directly neurotoxic (Benilova et al., 2020). Microglia deficiency did not lead to the increased accumulation of prions in the brain, suggesting that microglial degradation of prions (if it occurs) can be compensated by other cells such as reactive astrocytes. We conclude that the non‐redundant function of microglia is to moderate the harmful effects of dysregulated reactive astrocytes and/or to provide supportive factors to neurons (Sariol et al., 2020). Consistent with that interpretation, microglia can suppress astrocyte phagocytic activity and astrocytes are capable of complete, though slower, clearance of neurons in the absence of microglia (Damisah et al., 2020). Previous studies have used a CSF1R kinase inhibitor to infer the role of microglia in CNS prion disease and reported that overall expression of A1‐ and A2‐ reactive astrocyte‐associated transcripts in the brain was enhanced upon microglial depletion (Carroll et al., 2018; Carroll et al., 2020). However, use of CSF1R inhibitors can lead to partial depletion of microglia, impact other kinases (e.g. KIT, FLT3), cause localized microglial cell death and impact monocytes and macrophages outside the brain. So, the impacts on pathology should be interpreted with caution (Hume et al., 2020).

During the early stage of prion infection, the reactive astrocytes were more abundant in the brains of *Csf1r* ^ΔFIRE^ mice. Although there was no induction of A1 neurotoxic astrocyte‐associated genes, the reactive astrocytes displayed signs of enhanced engulfment of neuronal synapses. The observation of activated astrocytes engulfing synapses in the superior colliculus (G3) region of the brains of *Csf1r* ^ΔFIRE^ mice at 98 dpi with prions was coincident with the commencement of overt clinical signs in these mice at this time. These observations strengthen the hypothesis that loss of neuronal connectivity underlies neurological symptoms and precedes complete loss of neurons (Brown et al., 2001; Cunningham et al., 2003; Jeffrey et al., 2000). The engulfment of damaged synapses and neurons by reactive astrocytes could provide a clearance mechanism to protect surrounding undamaged neurons and synapses, as neuronal damage is required for astrocyte‐mediated toxicity (Guttenplan et al., 2020).

Independent studies have shown that the reactive astrocytes in the prion infected brain express complement component C3 and LCN2 highly (Hartmann et al., 2019; Kushwaha et al., 2021; Smith et al., 2020). In the current study the onset of the expression of C3 and LCN2 in reactive astrocytes was accelerated in the brains of infected *Csf1r* ^ΔFIRE^ mice compared to infected *Csf1r* ^WT^ mice. Further studies are required to determine whether complement component C3 and LCN2 contribute to the development of the neuropathology in the prion disease‐affected brain, or whether they are simply indicative markers of dysregulated reactive astrocytic activation. Hartmann and colleagues showed that the abolishment of C3+ astrocytes in mice deficient in TNFα, interleukin‐1α and complement component C1qa coincided with accelerated CNS prion disease (Hartmann et al., 2019). However, independent studies have shown that deficiency in complement component C3 does not affect the development of CNS prion disease (Klein et al., 2001), and TNFα was undetectable in the brains of prion‐infected *Csf1r* ^ΔFIRE^ mice. Studies from other CNS disorders suggest that the secretion of LCN2 from reactive astrocytes may contribute to the neuropathology by enhancing neuroinflammation or neurotoxicity (reviewed in Lim et al., 2021). The expression of LCN2 may also play a role in the phagocytosis of neuronal material by the reactive astrocytes (Wan et al., 2022).

The phosphorylated activation of PERK and eIF2α in the unfolded protein response pathway is also upregulated in reactive astrocytes during CNS prion disease (Smith et al., 2020), and the onset of this activation was similarly accelerated in the brains of microglia‐deficient prion‐infected *Csf1r* ^ΔFIRE^ mice. Targeted blockade of this pathway specifically in astrocytes has proved beneficial during prion disease (Smith et al., 2020). Our data from microglia‐deficient *Csf1r* ^ΔFIRE^ mice indicate that the microglia employ mechanisms to protect the neurons in the brain against prion infection by restricting both phagocytosis and unfolded protein response in astrocytes. A similar role for microglia has recently been described in the suppression of ATP‐mediated excitoxicity in neurons (Badimon et al., 2020).

In conclusion, our data indicate that the microglia provide neuroprotection independently of PrP^Sc^ clearance during prion disease and restrict the harmful activities of reactive astrocytes. Since astrocytes can contribute to both prion replication (Krejciova et al., 2017; Raeber et al., 1997) and synaptic loss in infected brains, preventing these activities would have therapeutic potential (Smith et al., 2020). Of course, since microglia have been attributed essential functions in CNS development and homeostasis (reviewed in Prinz et al., 2019) we cannot entirely exclude the possibility that the absence of microglia in *Csf1r* ^ΔFIRE^ mice may have rendered their neurons more vulnerable to prion‐mediated damage. However, CNS development appears normal in *Csf1r* ^ΔFIRE^ mice despite the complete absence of microglia (Rojo et al., 2019). We also cannot exclude the possibility that the microglia play a role in modulating prion particle toxicity. Abnormal prion accumulations within the brain may comprise a mixture of fibrillar and smaller oligomers of PrP^Sc^. However, since the smaller, non‐fibrillar, PrP^Sc^ particles are more pathological than larger fibrillary aggregates (Silviera et al., 2005), the engulfment and partial digestion of fibrillary PrP^Sc^ aggregates by the microglia may instead enhance their toxicity in the brain.

Further studies are now required to identify the molecular mechanisms by which the microglia provide neuroprotection during CNS prion disease. The previous characterization of the *Csf1r* ^ΔFIRE^ mice included mRNA expression profiling of the hippocampus which identified 85 transcripts that were significantly depleted when compare to wild‐type mice, and were presumably not compensated by astrocytes or other cells (Rojo et al., 2019). That list does not include most endosomal and lysosome‐associated genes that are more highly expressed by microglia and by inference must be upregulated by other cells in *Csf1r* ^ΔFIRE^ mice. An overlapping gene list was generated by expression profiling multiple brain regions in the *Csf1rko* rat (Pridans et al., 2018). Amongst the most down‐regulated transcripts are the three subunits of C1q, which have been implicated in regulating astrocyte function (Clarke et al., 2018; Liddelow et al., 2017) and neurodegeneration (Cho, 2019) and have complex roles in neuronal development and homeostasis (Vukojicic et al., 2019). These *Csf1r*‐dependent genes provide a short list of non‐redundant pathways that may be used by microglia to provide this neuroprotection and restrict the reactive astrocyte activation in prion disease. Paradoxically, given the focus of the literature on harmful functions of microglia, enhancing their functions may provide novel intervention treatments against these devastating neurodegenerative disorders.

## AUTHOR CONTRIBUTIONS

Barry M. Bradford, David A. Hume, Clare Pridans, and Neil A. Mabbott conceived the study; Neil A. Mabbott obtained funding; Barry M. Bradford and Neil A. Mabbott designed the experiments; Barry M. Bradford and Lynne I. McGuire performed the experiments and acquired data; all authors interpreted these data and contributed to the final version of this report.

## CONFLICT OF INTEREST

The authors declare no conflicts of interest.

## Data Availability

The data that support the findings of this study are available from the corresponding author upon reasonable request.
